# The Biological Characteristics of *Mycobacterium* Phage Henu3 and the Fitness Cost Associated with Its Resistant Strains

**DOI:** 10.3390/ijms25179301

**Published:** 2024-08-27

**Authors:** Xinyu Li, Junge Xu, Yuhan Wang, Salwa E. Gomaa, Huijie Zhao, Tieshan Teng

**Affiliations:** 1 Institute of Biomedical Informatics, School of Basic Medical Sciences, Henan University, Kaifeng 475004, China; 2023010033@henu.edu.cn (X.L.); 2023010014@henu.edu.cn (J.X.); wangyh@henu.edu.cn (Y.W.); 2 Department of Microbiology and Immunology, Faculty of Pharmacy, Zagazig University, Zagazig 44519, Egypt; salwaesmat@zu.edu.eg

**Keywords:** bacteriophage, *Mycobacterium smegmatis*, phage therapy, phage-resistant strain

## Abstract

Tuberculosis (TB), caused by *Mycobacterium tuberculosis*, is an infectious disease that seriously affects human life and health. Despite centuries of efforts to control it, in recent years, the emergence of multidrug-resistant bacterial pathogens of *M. tuberculosis* due to various factors has exacerbated the disease, posing a serious threat to global health. Therefore, a new method to control *M. tuberculosis* is urgently needed. Phages, viruses that specifically infect bacteria, have emerged as potential biocontrol agents for bacterial pathogens due to their host specificity. In this study, a *mycobacterium* phage, Henu3, was isolated from soil around a hospital. The particle morphology, biological characteristics, genomics and phylogeny of Henu3 were characterized. Additionally, to explore the balance between phage resistance and stress response, phage Henu3-resistant strains 0G10 and 2E1 were screened by sequence passage and bidirectional validation methods, which significantly improved the sensitivity of phage to antibiotics (cefotaxime and kanamycin). By whole-genome re-sequencing of strains 0G10 and 2E1, 12 genes involved in cell-wall synthesis, transporter-encoded genes, two-component regulatory proteins and transcriptional regulatory factor-encoded genes were found to have mutations. These results suggest that phage Henu3 has the potential to control *M. tuberculosis* pathogens, and phage Henu3 has the potential to be a new potential solution for the treatment of *M. tuberculosis* infection.

## 1. Introduction

Since the introduction of penicillin for treating bacterial infections, antibiotics have not only prevented and treated a variety of infectious diseases, but they have also revolutionized modern medicine by advancing clinical practices in surgery, organ transplantation, cancer chemotherapy and the use of artificial devices. However, the overuse of antibiotics has led to bacteria becoming less sensitive to these drugs due to natural selection and genetic mutations. Additionally, bacteria can transfer resistance genes to other bacteria through external DNA elements, such as plasmids and integrons [1]. In recent years, the occurrence of clinically multidrug-resistant and pan-resistant bacteria has notably increased, particularly with the advent of super-resistant strains. The rapid proliferation of bacterial drug resistance now poses a global health challenge, significantly heightening the risk of treatment failure and complications [2].

Tuberculosis (TB), caused by *M. tuberculosis* and primarily affecting the lungs, spreads through the air when an infected person coughs, sneezes or spits, with just a few inhaled germs needed to cause infection [3,4]. According to the WHO report, tuberculosis ranks among the top 10 causes of death worldwide, remains the second leading cause of death from a single infectious agent after COVID-19 and causes nearly twice as many deaths as HIV/AIDS [5]. Additionally, the number of TB patients resistant to rifampicin and isoniazid—the most effective anti-tuberculosis drugs—continues to rise, underscoring a critical situation in the resistance of *M. tuberculosis* [6,7,8]. Both multidrug-resistant TB (MDR-TB) and rifampicin-resistant TB (RR-TB) necessitate treatment with second-line drugs [9,10]. Treatment success rates stand at 88% for individuals with drug-susceptible TB but drop to 63% for those with MDR/RR-TB.

Phages provide innovative approaches for treating drug-resistant bacterial infections in humans due to their abundant sources [11], host specificity and superior bactericidal properties compared to antibiotics [12]. Used alone or in combination with antibiotics, phages offer significant antimicrobial benefits and hold wide-ranging application potential [13,14,15]. In recent years, there has been an increase in reports on the use of phages for treating *mycobacterium* infections, and the effectiveness of *mycobacterium* phages against various *mycobacterium* infections has been successfully demonstrated [16,17].

In this study, we isolated and characterized a strain of *mycobacterium* phage named Henu3. We analyzed its biological properties and genome to provide a theoretical foundation for the design of novel *M. tuberculosis* treatments using phage therapy. The goal of this research is to contribute to the development of future antimicrobial products and offer a new strategy for combating *M. tuberculosis*.

## 2. Results

### 2.1. Isolation and Morphological Characterization of Henu3

**Figure 1:**
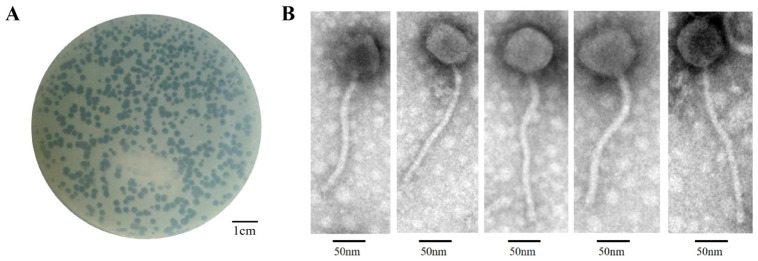
Morphological characterization of the Phage Henu3. (**A**) Phage plaques formed on the double layer agar plates; (**B**) Transmission electron microscopy photograph of phage particles.

*M. Smegmatis* mc^2^ 155, a nonpathogenic bacterium with a shorter colony formation cycle compared to *M. tuberculosis*, has frequently been used in place of the highly pathogenic and slow-growing *M. tuberculosis* in numerous molecular biology and molecular genetics experiments [18,19]. In this study, *M. smegmatis* mc^2^ 155 was used as a host for a new *mycobacterium* phage, Henu3, isolation from soil samples collected around a hospital environment. Phage Henu3 formed clear, circular plaques on the host bacterial lawn, measuring 4 to 6 mm in diameter with well-defined edges, indicating its lytic nature (Figure 1A). TEM characterization showed that phage Henu3 possesses a non-contractile tail measuring approximately 215 nm in length and an icosahedral head with an average diameter of 54 ± 3 nm (Figure 1B). Based on these morphological features and according to the latest International Committee on Taxonomy of Viruses (ICTV) guidelines (https://talk.ictvonline.org/ (accessed on 2 Apirl 2023)), phage Henu3 has been classified within the *Caudoviricetes* class [20].

### 2.2. Biological Characterization of Phage Henu3

**Figure 2:**
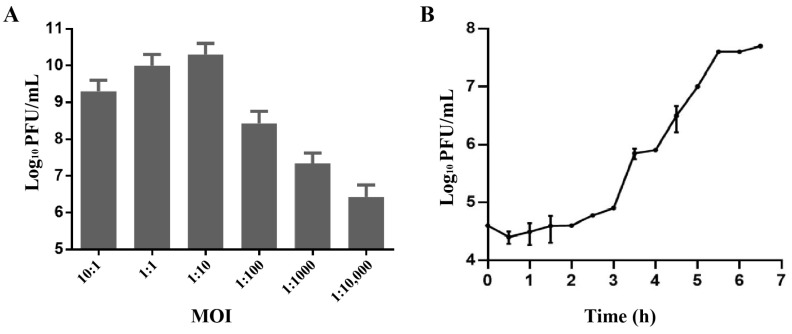
Biological characterization of phage Henu3. (**A**) The optimal multiplicity of infection (MOI) of phage Henu3; (**B**) One-step growth curve of phage Henu3 co-cultured with *M. smegmatis* mc^2^ 155. PFU, plaque forming unit. Error bars indicate standard deviation.

Phage Henu3 produced its highest phage titer of 3 × 10^10^ PFU/mL at a multiplicity of infection (MOI) of 1:10, indicating this to be the optimal MOI for phage Henu3 (Figure 2A). To further characterize the phage’s lifecycle, a one-step growth curve was performed using *Mycobacterium smegmatis* mc^2^ 155 at the optimal MOI. The phage Henu3 showed a latent period of 3 h, followed by a lytic period of 2.5 h. The average burst size was 40 PFU/cell (Figure 2B).

### 2.3. Influence of Temperature, pH, and Ultraviolet on Phage Henu3 Stability

**Figure 3:**
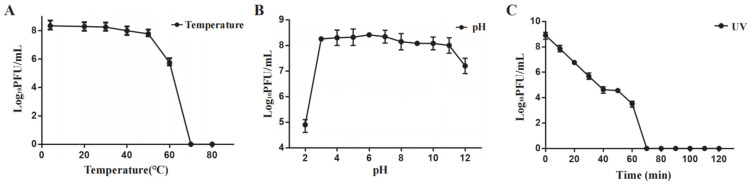
Stability testing of phage Henu3. (**A**) Thermal stability; (**B**) pH stability; (**C**) Sensitivity to ultraviolet light. Error bars indicate standard deviation.

Phage Henu3 stability was carried out under varying temperatures, pH range, and ultraviolet light. Phage Henu3 almost maintained its activity between 4 °C and 50 °C. A notable decline in phage titer was observed at 60 °C, whereas the phage titer was undetected at higher temperatures (Figure 3A). The acid-base tolerance test revealed that phage Henu3 was stable within a pH range of 3–11, with the highest titer observed at pH 7. The phage titer decreased tenfold in extremely alkaline conditions (pH 12) and thousandfold in highly acidic conditions (pH 2), suggesting that phage Henu3 is more tolerant to alkaline than acidic environments (Figure 3B). Moreover, under UV light exposure, the phage Henu3 titer progressively decreased over a duration of 60 min, with complete inactivation observed after 70 min, suggesting sensitivity to ultraviolet radiation (Figure 3C). Altogether, high temperatures (above 70 °C), pH levels outside the range of 3 to 11, or prolonged UV light exposure (70 min) can damage the genetic material of the phage, resulting in reduced infectivity and replication capability. Therefore, it is crucial to consider phage stability under various conditions when developing phage-based therapies to ensure their effectiveness.

### 2.4. The Whole-Genome Sequencing and Bioinformatics Analysis of Phage Henu3

**Figure 4:**
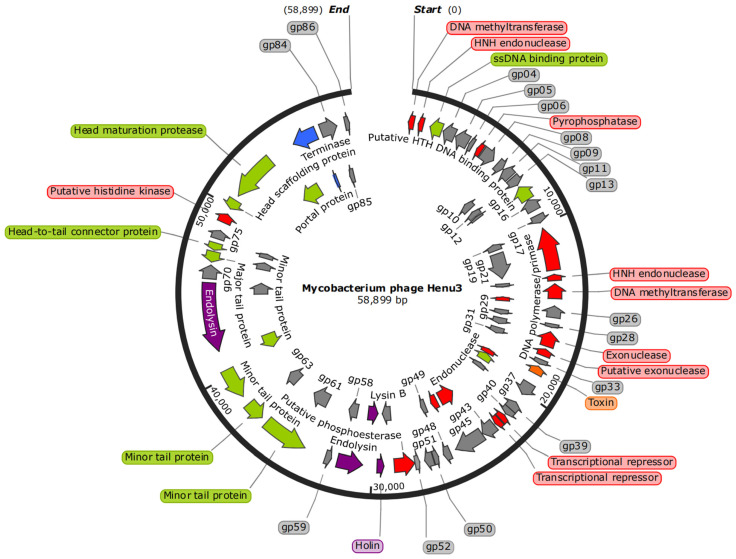
Complete Genome Map of mycobacteriophage Henu3. DNA metabolism (Red); Structure (Green); Lysis (Purple); Packaging (Blue); Hypothetical protein (Gray); Toxin (Orange).

The genome of phage Henu3 had a double-stranded DNA with 58,899 base pairs (bp) in length. The genomic composition is characterized by base contents of A (15.3%), G (32.6%), T (17.1%), and C (35%), resulting in GC content of 67.6% similar to that of its host, *M. smegmatis* mc^2^ 155, indicating a possible evolutionary adaptation to its host. The whole-genome sequence of mycobacteriophage Henu3 was deposited in GenBank (Accession Number MK224497). The phage Henu3 genome includes one tRNA gene (tRNA^Lys^) and 86 open reading frames (ORFs), with the ORFs encompassing 91.73% of the entire genome. These ORFs have an average length of 711 bp, distributed with 35 encoded in the forward direction and 51 in the reverse direction. Among these, 34 ORFs (39.5% of the total) have been identified with specific functions and are categorized into three functional groups: nucleic acid metabolism, DNA replication, and recombination; phage packaging and morphology; and proteins related to lytic functions (Figure 4).

**Figure 5:**
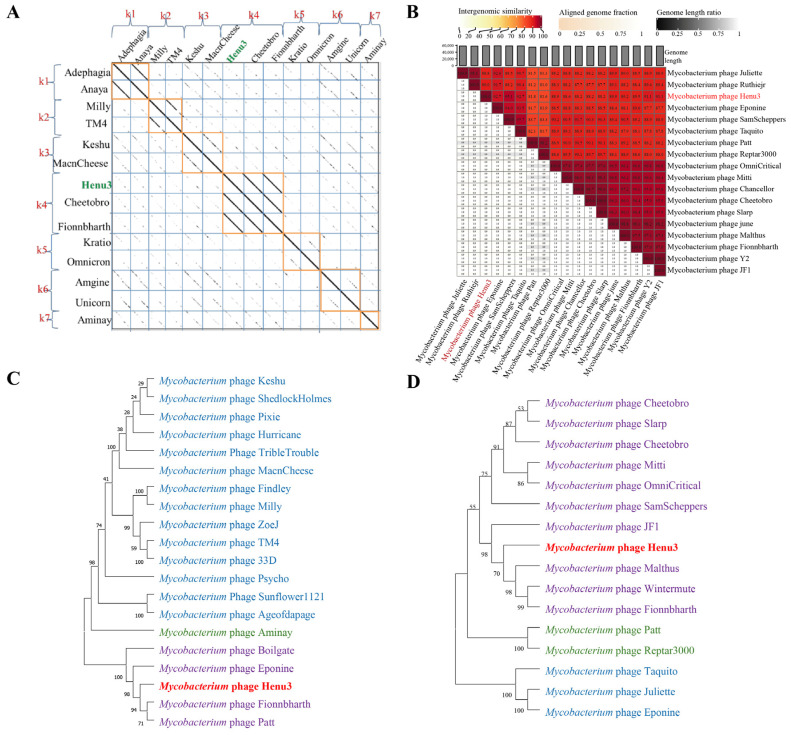
Dot-plot, genome-wide heatmap and evolutionary tree analysis of mycobacteriophage Henu3. (**A**) The dot-plot analysis of mycobacteriophage Henu3 highlights consecutive regions in the two sequences in orange; (**B**) Heatmap of Henu3 correlation with 17 phages. The right half of the heatmap shows the parasitic interphase of Henu3 with 17 mycobacteriophages; the more closely related the genome, the darker the color; (**C**) Phylogenetic tree indicating the relationship of Henu3 with tail proteins of other Mycobacteriophages; (**D**) Phylogenetic tree showing the relationship of Henu3 with tape measure proteins of other mycobacteriophages. Different colors represent distinct evolutionary branches, with Henu3 specifically highlighted in red.

**Figure 6:**
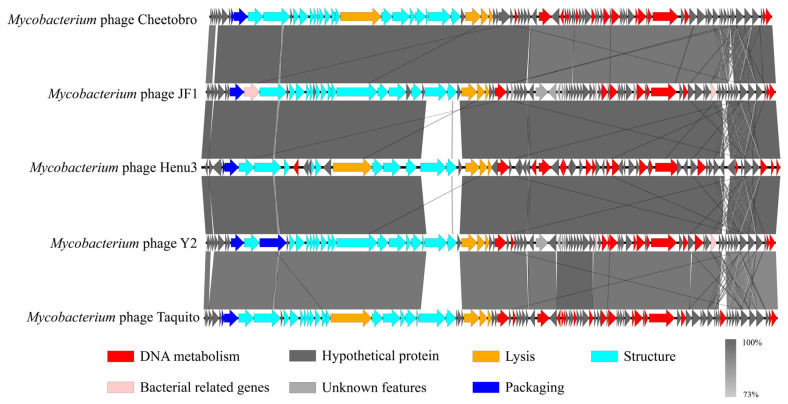
Genomic sequence similarity among Henu3, Cheetobro, JF1, Taquito and Y2. Predicted genes, transcription directions and associated functions are indicated by frameworks. Conserved regions are highlighted, with the color intensity representing the level of nucleotide sequence identity (ranging from 73% to 100%).

To elucidate the relationships of mycobacteriophage Henu3 with other phages, dot-plot analysis, heatmap analysis and phylogenetic trees’ construction were performed. The dot-plot analysis indicated the closest similarity between Henu3 and Fionnbharth within the *Mycobacterium* phage K4 sub-cluster (Figure 5A). When compared with other phages from the K cluster, the sequence coverage values for Henu3 were all below 40%, underscoring its distinctiveness and supporting its classification as a new member of the K4 sub-cluster. Heatmap analysis of genomic similarities revealed that the entire genome of Henu3 and other phages with greater than 80% blast query coverage on NCBI share significant likeness, including five phages that demonstrate over 90% similarity (Figure 5B). Phylogenetic trees constructed using the tail protein align Henu3 closely with mycobacteriophages Fionnbharth, Patt and Eponine (Figure 5C), while trees based on the tape measure protein indicate strong similarities between Henu3, Malthus, Wintermute and Fionnbharth (Figure 5D). Further comparative genomic analyses between Henu3 and previously documented mycobacteriophages (Cheetobro, JF1, Taquito and Y2) revealed that similarities at the amino acid level for DNA metabolism and key functional genes exceed 73% (Figure 6). Moreover, these phages also share highly similar assembly and lysis proteins, indicating common structural and functional characteristics.

### 2.5. Isolation of Phage-Resistant Bacteria

**Figure 7:**
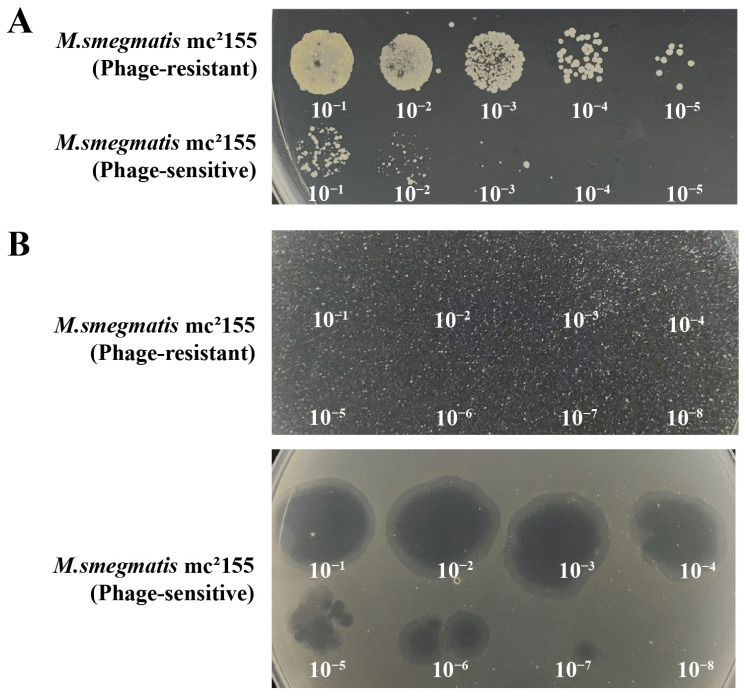
Verification of phage Henu3-Resistant Bacterial Strains. (**A**) The positive verification showed confluent growth of the phage-resistant strains and limited growth of the phage-sensitive strain; (**B**) The reverse verification exhibited no clear plaques in the phage-resistant strains, whereas visible clearing appeared in the phage-sensitive strain.

The phage-resistant strains, 0G10 and 2E1, were successfully isolated. During the forward verification procedure, these strains showed confluent growth on plates containing phage Henu3, unlike the phage-sensitive bacteria, which formed only a few colonies (Figure 7A). Phage-resistant strains have CFU counts in the order of 1.8 × 10^8^, while phage-sensitive strains have CFU counts in the order of 8 × 10^5^. Additionally, in the reverse verification procedure, when phage Henu3 was spotted onto 7H10 agar plates containing phage-resistant strains, no lysis was observed. In contrast, lysis zones were evident with the phage-sensitive bacteria, Henu3 in phage-sensitive strains exhibits PFU on the order of 1 × 10^9^ (Figure 7B). This indicates a successful selection of phage-resistant strains with stable resistance traits.

### 2.6. Phage-Resistant Strain Exhibits Increased Sensitivity to Antibiotics

**Figure 8:**
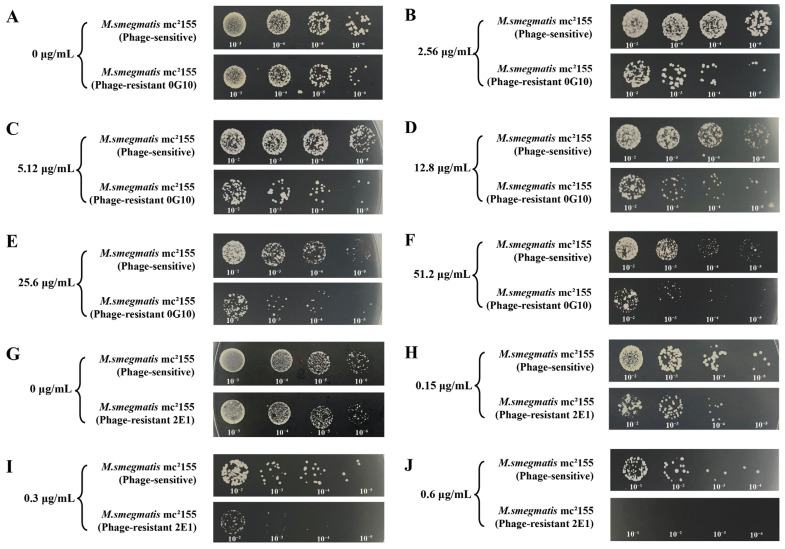
Susceptibility of phage-resistant and phage-sensitive strains to antibiotics. (**A**–**F**) Comparison of the sensitivity of phage-resistant strain 0G10 and phage-sensitive bacteria to varying concentrations of cefotaxime; (**G**–**J**) Sensitivity of phage-resistant strain 2E1 to different concentrations of kanamycin.

**Figure 9:**
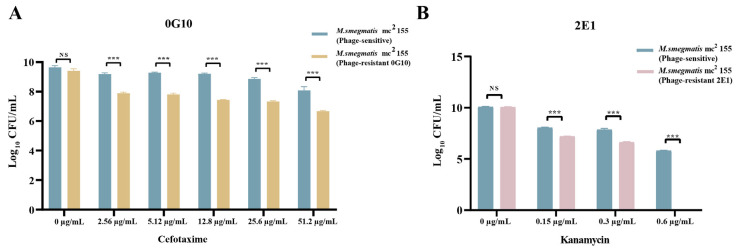
Susceptibility of phage-resistant and phage-sensitive strains to antibiotics. (**A**) CFU counts for phage-resistant strain 0G10 and phage-sensitive strains under various concentrations of cefotaxime. (**B**) CFU counts for the resistant and susceptible strain 2E1 at different concentrations of kanamycin. NS: not significant; ***: *p*-value < 0.001. Error bars indicate standard deviation.

The MIC values for cefotaxime against the phage-resistant (0G10) and sensitive strains were 84.6 µg/mL and 102.4 µg/mL, respectively. Both strains were grown in the presence of sub-MIC values of cefotaxime. The 0G10 strain displayed increased sensitivity to cefotaxime compared to its sensitive counterparts. Neither the 0G10 strain nor the phage-sensitive strain showed significant differences in CFU counts on plates without cefotaxime. However, at higher cefotaxime concentrations of 2.56 µg/mL, 5.12 µg/mL, 12.8 µg/mL, 25.6 µg/mL and 51.2 µg/mL, the CFU counts for the 0G10 strain were significantly lower than those for the sensitive strain (Figure 8A–F). The order of magnitude of CFU counts for phage-resistant (0G10) and sensitive strains corresponding to different concentrations of cefotaxime are shown in Figure 9A.

In addition, for kanamycin, the phage-resistant (2E1) and sensitive strains had MICs of 0.45 µg/mL and 1.2 µg/mL, respectively. Both phage-resistant 2E1 and sensitive strains showed identical CFU counts on kanamycin-free plates. However, when exposed to kanamycin concentrations of 0.15 µg/mL and 0.3 µg/mL, CFU counts for the 2E1 strain decreased by an order of magnitude compared to the phage-sensitive strain. Interestingly, at a higher concentration of 0.6 µg/mL, the 2E1 strain failed to show any colonies, indicating an extreme sensitivity to kanamycin in contrast to the phage-sensitive strain (Figure 8G–J). The magnitudes of CFU counts for phage-resistant 2E1 and sensitive strains, corresponding to different concentrations of kanamycin, are presented in Figure 9B.

### 2.7. Whole-Genome Re-Sequencing Analysis of Phage-Resistant Bacteria 0G10 and 2E1

To investigate the balancing act between phage resistance and antibiotic sensitivity in strains 0G10 and 2E1, whole-genome sequencing analysis was performed. A total of 12 insertion–deletion (InDel) mutations were identified when comparing these genomes with that of the sensitive strain (Table 1). Among these mutations, two were exclusive to the 2E1 genome, one was unique to 0G10, and the remaining nine mutations were common to both 0G10 and 2E1. These mutations affected genes encoding proteins associated with cell-wall synthesis enzymes, cell membrane transport proteins, transcriptional regulators, and two-component regulatory systems. One notable gene, MSMEG_2348, encodes a glycosyltransferase implicated in the glycosylation of the phospho-wall teichoic acid component of bacterial cell walls. Previous studies in *Streptomyces coelicolor* have shown that the absence of this glycosyltransferase enhances bacterial sensitivity to antibiotics, such as vancomycin and β-lactam antibiotics, and increases resistance to phage φC31 invasion [21]. Additionally, the genes MSMEG_5878, MSMEG_4512 and MSMEG_6720, which encode a keratinase, the mycobactin polyketide synthase MbtD, and a hydrolase, respectively, are involved in the synthesis of cell-wall peptidoglycan or phospho-wall teichoic acid. It is hypothesized that mutations in these four genes may alter phage adsorption mechanisms, thereby facilitating the emergence of phage-resistant bacterial strains.

In *M. smegmatis*, the genes MSMEG_0916, MSMEG_1060 and MSMEG_6084 encode the transcription factors TetR, Lsr2 and GntR, respectively. These factors regulate various cellular processes, including amino acid metabolism, DNA replication and repair. Lsr2, acting as a transcriptional repressor, directly controlimped gene expression by binding to promoter regions. Marta Kołodziej and colleagues showed that Lsr2 regulates the expression of mycobacterial polyketide synthase, which is crucial for synthesizing lipooligosaccharides. Mycobacteria lacking Lsr2 form smooth colonies and exhibit increased sensitivity to rifampicin and nalidixic acid [22]. Similarly, Kriti Arora and others have reported that Lsr2 deficiency impeded phage infection [23]. While it is hypothesized that other transcription factors might influence the antibiotic stress response, further experiments are required for confirmation. Additionally, genes MSMEG_0555, MSMEG_4468, MSMEG_2004 and MSMEG_6758 encode a sugar ABC transporter protease, a substrate-binding protein, an MFS family membrane transporter protein, and an aquaporin, respectively. Research has indicated that the absence of the substrate-binding protein DppA1 in *Pseudomonas aeruginosa* inhibits Pf5 phage adsorption and enhances biofilm formation [24]. It is speculated that similar membrane transport proteins in *M. smegmatis* may affect phage DNA entry and contribute to phage resistance.

The gene MSMEG_6236 encodes a two-component regulatory system (MnoR) commonly found in bacteria. This system, comprising a sensor histidine kinase and a response regulator, is crucial for bacteria to detect environmental changes, regulate the expression of growth and virulence factors, and adapt to stress. It functions through the phosphorylation and dephosphorylation of proteins. Lucas-Elío et al. demonstrated that such systems can regulate the expression of the CRISPR-Cas system, thereby playing a significant role in phage infection prevention. It is hypothesized that the MnoR system encoded by MSMEG_6236 might serve a similar function in mycobacteria [25], although further experimental validation is necessary.

## 3. Discussion

Due to the overuse of clinical antibiotics, there has been a surge in drug-resistant bacteria, pushing humanity towards a “post-antibiotic era” where effective antibiotics may no longer be available [26]. In response to this antibiotic crisis, relevant authorities have implemented a series of policy measures to restrict antibiotic use [27]. Phage therapy has emerged as a novel approach for treating antibiotic-resistant bacterial infections, offering a promising alternative to traditional antibiotics [28]. Phages offer significant advantages over antibiotics, including high specificity, safety, and a lower propensity for resistance development, thereby garnering considerable global scholarly interest.

In this paper, we isolated and sequenced the whole genome of a novel *mycobacterium* phage, henu3. Genomic and comparative genomic analyses revealed that it lacks virulence and endotoxin coding genes, indicating its promising potential for clinical applications at the molecular level. However, as phages are bacterial viruses, they must remain active under specific conditions, such as temperature and pH. Interestingly, we discovered that, compared to wild-type strains, the phage-resistant strains 0G10 and 2E1 exhibited increased sensitivity to the antibiotics kanamycin and cephalosporin, respectively. This suggests that bacterial resistance to phages may not be a significant factor affecting the clinical application of phage therapy. Currently, the clinical application of phages faces several significant challenges. For instance, with *mycobacterium* phages, not all bacteriophages capable of infecting *M. smegmatis* will necessarily infect *M. tuberculosis* or other *Mycobacterium* [29]; validating their cross-infection capability is therefore a prerequisite for their use. Furthermore, an additional major obstacle with *M. tuberculosis* is that it is an intracellular pathogen [10], and how phages can penetrate the eukaryotic cell membrane to reach and kill the intracellular bacteria poses a new challenge.

Overall, phage therapy holds considerable promise, with successful instances of using phages to treat *Mycobacterium abscessus* infections post-surgery already documented [16,30]. The development of appropriate phage cocktails and the strategic modification of specific phage genes are crucial for the success of clinical phage treatments. Additionally, the application of phage-based hydrogels in treating skin burns [31,32] and inhaled phages for respiratory bacterial infections [33] has demonstrated substantial potential. However, preclinical studies involving phages need to be further strengthened, including experimental treatments of animal infection models with phages, despite the fact that such research demands higher laboratory standards due to the virulence of some infectious bacteria.

## 4. Materials and Methods

### 4.1. Isolation and Purification of Phage Henu3

Phage was isolated from the soil sample collected from the hospital of the first affiliated hospital of Henan University. Approximately 15 g of soil from the hospital surroundings were dissolved in 30 mL of SM buffer and subsequently filtered through a 0.22 µm filter to remove bacteria. A total of 2 mL of log-phase *M. smegmatis* mc^2^ 155 was added to a conical flask containing 30 mL of the filtrate and incubated in a thermostatic shaker at 37 °C for three days. The mixed culture was then centrifuged at 12,000 rpm for six minutes to precipitate the bacteria, and the supernatant was further filtered through a 0.22 µm filter. The presence of phage in the filtrate was screened using the spot assay. The isolated phage was purified using the double-layer agar (DLA) technique [34,35], which was repeated ten times to ensure consistency in plaque morphology.

### 4.2. Transmission Electron Microscopy (TEM)

Phage Henu3 morphology was examined using TEM. Briefly, 20 μL of purified phage lysate was placed onto a copper grid and allowed to stand for 15 min. Excess liquid was removed using filter paper, and the grid was then stained with 2% phosphotungstic acid for 10 min before visualizing the phage particles using a Tecnai 10 (Philips, Amsterdam, The Netherlands) TEM [36].

### 4.3. Genomic Sequencing of Phage Henu3

Phage Henu3 genomic DNA was extracted using a combination of Proteinase K-Sodium dodecyl sulfate (SDS), and phenol extraction methods [37]. The extracted DNA was then sequenced using next-generation sequencing technology on the Illumina 2100 platform. The genome was fragmented using ultrasonication (Fisher Scientific, Hampton, NH, USA), generating randomly sized genomic fragments. For DNA library construction, fragments approximately 300 bp in length were isolated using gel electrophoresis and purified with the TruSeq DNA Sample Prep Kit-Set A. Libraries were then amplified using the TruSeq PE Cluster Kit (Illumina, San Diego, CA, USA) and sequencing reactions were conducted on an Illumina sequencer. For sequence assembly, data were compiled using Newbler 3.0 software to reconstruct the complete phage genome sequence [38].

### 4.4. Bioinformatics Analysis of the Whole-Genome Sequence of Henu3

The phage genome sequences were characterized using EditSeq from the DNASTAR package, with analyses conducted on the base composition, G + C content, and the average gene length and density across the genome. The Open Reading Frames (ORFs) were predicted using Softberry (http://www.softberry.com/ (accessed on 8 May 2023)) and GeneMark [39] (https://genemark.bme.gatech.edu/GeneMark/ (accessed on 10 May 2023)), selecting ATG, TTG, and GTG as start codons. The complete Genome Map of *Mycobacterium* phage Henu3 is based on Henu3 whole-genome sequencing data and constructed by SnapGene 7.1.0 [40]. The dot-plot analysis was performed with the Gepard tool (https://genskew.csb.univie.ac.at/ (accessed on 26 June 2023)) [41]. For phylogenetic analysis, a neighbor-joining tree was constructed using MEGA11 [42] with 1000 bootstrap replications, and phage family classification was performed using DNAplotter 17.0.1. Multiple genome comparisons were conducted with EasyFig 2.2.5 [43]. The heatmap of Henu3 correlation with 17 phages was mapped with the help of the VIRIDIC tool (https://rhea.icbm.uni-oldenburg.de/viridic/ (accessed on 13 February 2024) [44].

### 4.5. Optimal Multiplicity of Infection (MOI) for Phage Henu3

The optimal MOI of phage Henu3 was assessed as previously described [29]. In brief, *M. smegmatis* mc^2^ 155 in log-phase culture (2 × 10^7^ CFU/mL) was mixed with phage Henu3 at different MOI ratios of 10:1, 1:1, 1:10, 1:100, and 1:1000, respectively [45]. The phage-bacteria co-culture was incubated at 37 °C for 12 h in a shaking incubator, and the phage titer was determined using the DLA agar technique. The MOI that resulted in the highest titer was identified as the optimal MOI for phage Henu3. Each MOI was tested in triplicate to ensure accuracy.

### 4.6. One-Step Growth Curve of Phage Henu3

A one-step growth curve was conducted to determine the latent period and burst size of phage Henu3 [29,35]. Briefly, *M. smegmatis* mc^2^ 155 log-phase culture and phage Henu3 was mixed at the optimal MOI ratio (1:10) and incubated at 37 °C for 30 min. Following incubation, the mixture was centrifuged, and the supernatant was discarded. The pellet was re-suspended in 10 mL of 7H9 medium and re-incubated. Samples were taken every 30 min for phage Henu3 titer determination by the DLA technique [35].

### 4.7. Thermal, pH, and Ultraviolet Stability of Phage Henu3

Phage stability was evaluated against various conditions as previously described [46]. In brief, a highly concentrated phage solution (1 × 10^9^ PFU/mL) was incubated for 1 h at different temperatures (4 °C, 20 °C, 30 °C, 40 °C, 50 °C, 60 °C, 70 °C and 80 °C) or under varying pH values (2, 4, 6, 8, 10 and 12) for 1 h at 27 °C. After incubation, the phage titer was determined using the DLA technique. To evaluate ultraviolet (UV) stability, EP tubes containing phage solution were positioned under a 40 W UV lamp at a wavelength of 253.7 nm, with the distance between the tubes and the lamp set to 10 cm. Aliquots of 100 µL were withdrawn every 10 min for titer determination.

### 4.8. Testing for Phage-Resistant Strains

The phage-resistant strains were screened using the traditional spot assay [47,48,49]. The bacterial colonies that developed within the lysis zones were picked up and underwent two rounds of streaking onto 7H10 agar plates to obtain pure individual colonies. The phage-resistant colonies were further confirmed using bidirectional validation techniques. For the forward validation procedure, both phage-resistant or phage-sensitive *M. smegmatis* mc^2^ 155 strains were tenfold serially diluted, and aliquots of 5 µL of each diluted culture were inoculated onto the surface of 7H10 agar plates containing Phage Henu3 in the top soft agar layer. Following incubation at 37 °C for 72 h, the plates were inspected for the phage-resistant colonies. For reverse validation procedure, phage Henu3 solution (1 × 10^9^ PFU/mL) was serially diluted, and 5 µL aliquots from each dilution were spotted onto 7H10 agar plates pre-seeded with either phage-resistant or phage-sensitive *M. smegmatis* mc^2^ 155 strains, respectively. No lysis zones following incubation at 37 °C for 48 h confirms the phage-resistance phenotypes [47]. The entire procedure was repeated three times to ensure consistent results.

### 4.9. Forward and Reverse Validation of Phage-Resistant Bacteria

Positive Validation Procedure: 300 µL of high-concentration phage solution (1 × 10^9^ PFU/mL) was evenly spread on a 7H10 agar plate. Both resistant (phage-resistant *M. smegmatis* mc^2^ 155) and sensitive (*M. smegmatis* mc^2^ 155) bacterial strains were serially diluted in tenfold increment using the same high-concentration phage solution and MP buffer, respectively. Subsequently, 5 µL of each diluted culture—both phage-resistant and sensitive—was dropwise added to a 7H10 plate containing phage Henu3. The plates were then incubated in a biochemical incubator at 37 °C for 72 h to assess the number of CFUs. This test was performed three times to ensure consistency in the results.

Reverse Validation Procedure: double-layer agar plates were prepared with both phage-resistant and sensitive *M. smegmatis* mc^2^ 155 strains. Concurrently, phage Henu3 at a concentration of 1 × 10^9^ PFU/mL was serially diluted from 10^−1^ to 10^−8^. Subsequently, 5 µL of Phage Henu3 from each dilution gradient was added dropwise onto the respective double-layer agar plates. The plates were allowed to dry and then incubated in a biochemical incubator at 37 °C for 48 h. For plates with resistant bacteria, no plaques (empty spots) are expected, whereas plates with sensitive bacteria should show clear plaque formation. The number of plaques at different dilution gradients was observed and recorded. This procedure was repeated three times to ensure consistent results.

### 4.10. Detection of Susceptibility of Phage-Resistant Bacteria to Various Antibiotics 

The minimum inhibitory concentration (MIC) of cefotaxime and kanamycin was determined against the phage-sensitive and phage-resistant *M. smegmatis* mc^2^ 155 strains using the broth microdilution method [50]. Subsequently, the phage-resistant *M. smegmatis* mc^2^ 155 strains were grown in 7H9 medium in the presence of 1/2 MIC, 1/4 MIC, 1/8 MIC, and so forth. In brief, aliquots of 195 µL of 7H9 medium with varying antibiotic concentrations were dispensed into each well of the microtiter plate. Then, aliquots of 5 µL of phage-resistant bacteria in the log-phase were added to each well. The plates were then incubated at 37 °C for 72 h. The phage-sensitive *M. smegmatis* mc^2^ 155 strain served as a control. Samples were taken every 12 h for colony forming unit (CFU) counts. This procedure was performed in triplicate to ensure consistency in the results.

### 4.11. Statistical Analysis

The experiments were replicated three times, and the experimental results are expressed as mean ± standard deviation (SD). All results were analyzed by multiple *t* tests for a significance level of *p* < 0.05. GraphPad Prism 9.0 (GraphPad Software, San Diego, CA, USA) was used for data analysis.

## Figures and Tables

**Table 1:** Information on mutation sites of phage Henu3-resistant bacteria.

Strain	Mutation Location	Mutant Genes	Type of Mutation	REF	ALT	Function
2E1	632863	MSMEG_0555	INDEL	C	CACACTGTTCTGCGCGTTGTAGACGCGGTTCGACGACGCCGACAGCTCTTTGT	Carbohydrate ABC transporter permease
0G102E1	1000473	MSMEG_0916	INDEL	GCC	G	TetR/AcrR family transcriptional regulator
0G10, 2E1	1127759	MSMEG_1060	INDEL	CCTG	C	Lsr2 family protein
0G10, 2E1	2085173	MSMEG_2004	INDEL	CGG	C	MFS transporter
0G10	2429903	MSMEG_2348	INDEL	C	CG	Glycosyltransferase
2E1	4550626	MSMEG_4468	INDEL	GAAT	G	Sugar ABC transporter substrate-binding protein
0G10, 2E1	4596508	MSMEG_4512	INDEL	GC	G	Mycobactin polyketide synthase MbtD
0G10, 2E1	5940372	MSMEG_5878	INDEL	C	CGG	Cutinase family protein
0G10, 2E1	6148093	MSMEG_6084	INDEL	G	GC	Helix-turn-helix domain containing protein
0G10, 2E1	6302176	MSMEG_6236	INDEL	T	TGGCCTC	Two-component system response regulator MnoR
0G10, 2E1	6768692	MSMEG_6720	INDEL	GC	G	Alpha/beta hydrolase
0G10, 2E1	6803027	MSMEG_6758	INDEL	GCACCCT	G	Aquaporin family protein

## Data Availability

Complete genome of *mycobacterium* phage Henu3 is available in https://www.ncbi.nlm.nih.gov/nuccore/ (accessed on 21 January 2023) under the GenBank accession number MK224497.
